# Correlation Between Hypothyroidism and Gallstone Disease in Central India

**DOI:** 10.7759/cureus.56799

**Published:** 2024-03-23

**Authors:** Varun Kulkarni, Harshal Ramteke, Yashwant Lamture, Tushar Nagtode, Pankaj Gharde, Venkatesh Rewale

**Affiliations:** 1 Department of General Surgery, Jawaharlal Nehru Medical College, Datta Meghe Institute of Higher Education and Research, Wardha, IND

**Keywords:** gallbladder stones, gallbladder diseases, thyroid hormones, thyroid disorders, cholelithiasis

## Abstract

Introduction

The decreased thyroid-stimulating hormone (TSH) levels affect almost every nucleated cell in our body, which directly affects the basal metabolic rate (BMR). It tends to affect multiple organ systems in the human body. In recent times, there have been changes in lifestyle and the consumption of processed foods has increased. Thus, cholelithiasis and hypothyroidism are being encountered frequently, even in rural populations. In recent times, the overt clinical presentation of hypothyroidism is rare due to early diagnosis and treatment of the subclinical hypothyroidism state itself.

Aim

The aim is to determine the correlation between cholelithiasis and hypothyroidism.

Methods

This was a cross-sectional study done on the patients presenting to the surgical department at Acharya Vinoba Bhave Rural Hospital affiliated to Jawaharlal Nehru Medical College, Sawangi (Meghe), Wardha, Maharashtra, India between the duration from December 2020 to December 2022, having gallstone disease or with symptoms suggestive of gall bladder stones. The admitted patients were then evaluated for hypothyroidism by thyroid profile tests, and the results were documented. Using the collected data, factors such as clinical spectrum, correlation between cholelithiasis and hypothyroidism, relation with the demographic data, and body mass index (BMI) were studied.

Results

A total of 52 gallstone patients were included in the study. A greater number of patients were found to be above the age of 40 years (82.7%), with female preponderance (61.5%). More patients with cholelithiasis were from the group with having BMI more than normal (57.6%). More patients having both cholelithiasis and hypothyroidism were also from the group with having BMI more than normal. Most symptomatic patients complained of pain in the right hypochondriac region (88.5%). Of these 52 patients, nine (17.3%) were found to have hypothyroidism (seven were subclinical, two patients had overt clinical symptoms and signs) and the remaining 43 patients were euthyroid.

Conclusions

Our study supports that there is a correlation between cholelithiasis and hypothyroidism. Out of all the 52 patients, nine had hypothyroidism, seven were subclinical, and two had overt symptoms. Thus, we conclude that there is a correlation between cholelithiasis and hypothyroidism.

## Introduction

Cholelithiasis as a medical condition has been known to humans since ancient times. Gallstones were found in the mummified tombs of one of the priestesses of Amen during the 21^st^ Egyptian dynasty (around 1000 BC). That is when this disease was first diagnosed in humans. Cholelithiasis is a condition that is common worldwide. It is usually secondary to an abnormality in lipid and bile salt levels, which causes accumulation of the bile salts in the gallbladder cavity. Gallstones are the most common biliary tree disease, both in India and in Western countries. Gallstones affect around 10%-12% of adults in Western countries [1,2]. According to a survey on gallstones, north Indians have had gallbladder stones more frequently than South Indians [3]. 8% to 16% of gallstone patients also have stones in the common bile duct (CBD) [3]. The occurrence of pure cholesterol stones is uncommon. The most frequent initial process for the formation of cholesterol stones is the oversaturation of bile with cholesterol. Although phospholipid or bile salt secretion cannot produce supersaturation, hypersecretion of cholesterol nearly always does. Pigment stones have a low cholesterol content of under 20% and are blackish which is because of the high content of calcium bilirubinate.

For many years, there has continued a debate about whether the conditions of the thyroid gland and hormones can give rise to diseases of the gallbladder. The potential link between gallstone diseases and hypothyroidism may have numerous causes. The action of hormones secreted by the thyroid gland on cholesterol metabolism is well-established [4]. Increased serum cholesterol levels in hypothyroidism cause bile to become supersaturated with cholesterol, which impairs filling, decreases contractibility, and prolongs the time bile can stay in the gallbladder and flow through it [5-7]. These circumstances help keep cholesterol crystals in place, giving them enough time to form and continue to grow and lead to the maturation of gallstones [5]. Apart from this the decreased amount of bile secretion can physically hinder the removal of accumulated substances from the gallbladder and biliary ducts [8]. Additionally, the sphincter of Oddi (SO) is said to carry receptors for thyroid hormones. Thyroxine (T4) directly promotes sphincter relaxation.

Recent research has focused on the relationship between thyroid hormones, specifically triiodothyronine (T3) and thyroxine (T4), and gallstone formation. About 90% of the patients having hypothyroidism show high levels of cholesterol, and people with hypothyroidism have serum cholesterol levels that are roughly 50% greater than those of euthyroid patients [9]. Similarly, low T4 levels act on the feature of the SO to land up into a relaxed state, causing cholestasis and stone development [10]. Ultrasonography (USG) alone reliably detects bile duct stones in approximately 80%-90% of instances [10]. It is thus considered to be the first line and a reliable investigation for the diagnosis of gallstones.

The possible correlation of the hypothyroid state and cholelithiasis is yet to be extensively studied. Most of the studies that were conducted in the past on this topic were based on an urban population. However, there were no such detailed studies based particularly on the rural population. In recent times, the overt clinical presentation of hypothyroidism has been rare, but subclinical hypothyroidism is more frequently encountered. So, we carried out this study to assess the possibility of a correlation between hypothyroid state and cholelithiasis in a rural-based population.

## Materials and methods

Objectives and aim of the study

This study is based on determining the correlation between cholelithiasis and hypothyroidism. The objectives were, to estimate the frequency of patients with cholelithiasis who also had hypothyroidism and determine the relation between obesity and body mass index (BMI) with hypothyroidism and cholelithiasis.

Methodology

Type and Place of Study

It was a cross-sectional study, performed at Acharya Vinobha Bhave Rural Hospital, affiliated to Jawaharlal Nehru Medical College under Datta Meghe Institute of Higher Education and Research, Sawangi (Meghe) Wardha, Maharashtra, India.

Study Approval and Study Population

Before the commencement of this study - the Institutional Ethics Committee of Jawaharlal Nehru Medical College, Sawangi (Meghe), Wardha, had approved the study. Institutional Ethics Committee (IEC) number: DMIMS(DU)/IEC/2020/21/9410. Patients above 12 years of age with cholelithiasis were selected for the study within the duration starting from December 2020 to December 2022 (two years).

Sample Size

52 cases

$n = \frac{\frac{}{}z_{\frac{\alpha }{2}}^{2} . P . (1-P)}{E^{2}}$

Where, $Z_{\frac{\alpha }{2}}$ is the level of significance at 5%, i.e., 95% confidence interval = 1.96

P = Incidence of hypothyroidism = 16% = 0.16 E = Error of margin = 10% = 0.10

By this formula,

𝑛 = 51.63

𝑛 = 52 patients needed in this study.

Patients were selected by the purposive sampling method.

Inclusion criteria and exclusion criteria

Patients of cholelithiasis above 12 years of age, who came to the outpatient department (OPD) in the surgery department were included in our study. Children with ages less than 12 years were excluded from our study. Patients giving a history of any surgical intervention in the past involving the thyroid region, who were consuming any thyroid stimulating/antithyroid medications, with a history of taking contraceptive medications, and patients who underwent any radiation exposure were also excluded from our study.

Study procedure and ethical consideration

All patients having symptoms of diseases of gallstones hospitalized in the Department of General Surgery underwent a thorough physical examination, and clinical history was elicited after admission. Imaging scans were performed after basic investigations. Patients with evidence of cholelithiasis on the radiological investigations were chosen by random sampling method. In each case, a comprehensive physical examination was conducted. The investigator filled out the data collection forms. With a thyroid function test, patients diagnosed with cholelithiasis in the form of microlithiasis, a single gall bladder stone, or several stones in the gall bladder were checked for any thyroid disorders. The serum thyroid hormone (T3, T4, and thyroid-stimulating hormone (TSH)) levels were all evaluated as part of the thyroid function test carried out while the subject was fasting. Thyroxine supplementation therapy was started for those who were discovered to be hypothyroid.

All patients and legal guardians received a description of the study, the investigations, their benefits and drawbacks, anticipated outcomes, and potential side effects. If the subject concurred, the case had been chosen for this study. There were no extra investigations or substantial risks to the patient in this trial. The financial burden on the patient was not increased by it. The institutional ethical committee gave its prior approval to the project before data collecting could begin. All patients and their guardians gave their informed consent.

Collection of the data and analysis

Data were gathered using a pre-set questionnaire about the medical and surgical history of the patient and the symptoms pointing towards cholelithiasis and hypothyroid disorder. Data were gathered from all the patients by direct questioning them about their medical history after obtaining written informed consent from them and as and when needed from their legal guardians. Both manual and computer-based analysis of the data were conducted. A statistical package for social science (SPSS) was used, calculated data were organized systematically and displayed in various figures and tables, and a statistical analysis was conducted to assess the goals of our study. Data were coded and a Microsoft Excel spreadsheet program was used to keep track of the data. To analyze the data, the SPSS v23 (IBM Corp., Armonk, NY) program was employed.

## Results

Demographic profile

In our study, less number of patients were from the age group 12-40 years, and more patients were above the 40 years of age mark. Whereas the study showed, prevalence in female patients was more as compared to male patients. Our study shows a male (M):female (F) ratio of 1:1.6 (Tables 1, 2).

**Table 1:** Distribution of the patients in accordance with age group

Age Group	Frequency	Percentage
12-40 Years	9	17.3
>40 Years	43	82.7
Total	52	100

**Table 2:** Distribution of patients according to gender

Gender	Percentage	Frequency
Male patients	38.5	20
Female patients	61.5	32
Total patients	100	52

Distribution of cholelithiasis patients according to BMI

In our study, we found out that the maximum number of patients, 23, fell into the group having a BMI of 25.0-29.9 kg/m² which is borderline obese. Thirteen patients had a BMI of 23.0-24.9 kg/m² which was labeled as overweight. Eight patients had a BMI of 18.5-22.9 kg/m², which is normal. Five patients had a BMI of 30.0- 34.9 kg/m², severely obese. Two patients were morbidly obese, having a BMI of 35.0-39.9 kg/m². One patient had a BMI <18.5 kg/m² and thus was underweight (Table 3).

**Table 3:** Distribution of cholelithiasis patients in relation to BMI

BMI (Kg/m^2^)	Frequency	Percentage
<18.5	1	1.9
18.5-22.9	8	15.4
23.0-24.9	13	25.0
25.0-29.9	23	44.2
30.0-34.9	5	9.6
35.0-39.9	2	3.8
Total	52	100

Association between thyroid status and BMI

In the euthyroid patients, one of the participants had a BMI <18.5 kg/m². Eight participants had a BMI of 18.5-22.9 kg/m². Twelve participants had a BMI of 23.0-24.9 kg/m². Nineteen of the participants had a BMI of 25.0-29.9 kg/m². Three participants had a BMI of 30.0-34.9 kg/m².

Of all the patients with hypothyroidism, zero of the participants had a BMI <18.5 kg/m². Zero participants had a BMI of 18.5-22.9 kg/m². One of the participants had a BMI of 23.0-24.9 kg/m². Four of the participants had a BMI of 25.0-29.9 kg/m². Two of the participants had a BMI of 30.0-34.9 kg/m². Two of the participants had a BMI of 35.0-39.9 kg/m².

The overall distribution of the patients according to BMI shows that a higher number of hypothyroidism patients were obese. Strength of association between the two variables (Cramer's V) = 0.52 (High Association). Fisher's exact test was used to explore the association between “Thyroid Status” and “BMI” as more than 20% of the total cells had an expected count of less than 5. There was a significant difference between the various groups regarding the distribution of BMI (χ2 = 14.078, p = 0.029). The strength of association between the two variables (Bias Corrected Cramer's V) = 0.42 (Moderate Association) (Table 4).

**Table 4:** Association between thyroid status and BMI

BMI (kg/m^2^)	Thyroid Status			Fisher's Exact Test	
	Euthyroid	Hypothyroid	Total	χ2	P-value
<18.5	1 (2.3%)	0 (0.0%)	1 (1.9%)	14.078	0.029
18.5-22.9	8 (18.6%)	0 (0.0%)	8 (15.4%)		
23.0-24.9	12 (27.9%)	1 (11.1%)	13 (25.0%)		
25.0-29.9	19 (44.2%)	4 (44.4%)	23 (44.2%)		
30.0-34.9	3 (7.0%)	2 (22.2%)	5 (9.6%)		
35.0-39.9	-	2 (22.2%)	2 (3.8%)		
Total	43 (100.0%)	9 (100.0%)	52 (100.0%)		

Distribution of cholelithiasis patients according to the thyroid status

Our study studied the distribution of cholelithiasis patients as per the thyroid status according to the thyroid profile test. We found that, out of the 52 participants, 43 were euthyroid, and nine had hypothyroidism (Table 5).

**Table 5:** Distribution of cholelithiasis patients in accordance to the thyroid status

Thyroid Status	Frequency	Percentage
Euthyroid	43	82.7
Hypothyroid	9	17.3
Total	52	100

## Discussion

In the present study, 52 patients with cholelithiasis were randomly chosen and enrolled in the Department of General Surgery at Jawaharlal Nehru Medical College. The study's objectives were to determine the prevalence of hypothyroidism in patients with cholelithiasis, evaluate its significance as a causative factor, and integrate thyroid function tests into the usual workup of individuals with gallstones.

Demographic profile

In our study, nine patients (17.3%) were from the age group 12-40 years, and 43 patients (82.7%) were above the age of 40 years. In their research (sixth decade), Herman et al. have documented a comparable frequency [11]. Shaffer et al. performed an extensive survey, including patients from all over the world, and concluded that, as you age, the likelihood of developing gallstones increases [12]. Age causes a decrease in bile acid production, an increase in biliary cholesterol production, and an increase in cholesterol saturation. The bile acid synthesis rate-limiting enzyme, cholesterol 7a-hydroxylase (CYP7A1), is less active, which results in these negative effects [13,14]. Additionally, as people age, more risk factors may build up, resulting in lithogenesis [15]. Most of the patients whose data was obtained were in the 36 to 50-year range, with a mean patient age of 43 years [16]. Sadaf et al., in their study, mentioned that out of 100 patients, 40 were below 40 years, whereas 60 were above the age of 40 years [17]. However, the age distribution of the research showed an equal distribution of patients in both age groups in their study (Table 6) [16].

**Table 6:** Distribution of the patients according to age in various studies

Study	Number of patients in younger age group (<40 years)	Percentage of patients in younger age group	The younger age group of the study	Number of patients in elderly age group (>40 years)	Percentage of patients in older age group	The older age group of the study	Total
Present study	9	17.3	12-40 years	43	82.7	>40 years	52
Ghadhban et al. (2019) [18]	14	13.6	20-35 years	89	86.4	36-65 years	103
Herman et al. (1989) [11]	36	12.8	10-40 years	246	87.2	41-90 years	282
Sadaf et al. (2021) [17]	40	40	<40 years	60	60	>40 years	100
Yaser et at. (2018) [16]	72	48	<40 years	78	52	>40 years	150

In our study, 20 (38.5%) patients were males, whereas 32 (61.5%) were females who suffered from cholelithiasis. The M: F ratio is 1:1.6. Numerous studies confirmed what we found in our research: females had a higher prevalence of cholelithiasis than males [19-22]. Because of sex hormones and pregnancy, women are thought to be at higher risk, and traditional epidemiologic studies corroborate this. Estrogen causes bile to become hypercholesterolemic and lithogenic by increasing the release of biliary cholesterol [21]. Ghadhban et al. stated that 84 (81.6%) of the patients who underwent testing were female, and 19 (18.4%) were male showing a 1:5 male-to-female ratio [18]. Sarda et al., Raju et al., and Sachdeva et al. showed similar results as our study showing slight preponderance with the female gender in the incidence of cholelithiasis [19-21]. The studies by Hassan et al., Singh et al., and Ghadhban et al. showed a strong association between the female gender and the prevalence of cholelithiasis [16,18,22] similar to our study (Table 7).

**Table 7:** Distribution of patients in accordance with gender in various studies

Study	Number of male patients	Percentage of male patients	Number of female patients	Percentage of female patients	Total
Present study	20	38.5	32	61.5	52
Sarda et al. (2018) [19]	25	31.25	55	68.75	80
Raju et al. (2019) [20]	36	40	54	60	90
Sachdeva et al. (2011) [21]	59	39	91	61	150
Yaser et al. (2018) [16]	30	20	120	80	150
Singh et al. (2001) [22]	4	12.9	27	87.1	31
Ghadhban et al. (2019) [18]	19	18.4	84	81.6	103

Distribution of cholelithiasis patients according to BMI

In our study, we found out that, the most significant number of patients, 23 (44.2%), fell into the group who had a BMI of 25.0-29.9 kg/m² which is borderline obese. Thirteen (25.0%) patients were found to have a BMI of 23.0-24.9 kg/m² which was labeled as overweight. Eight (15.4%) patients had a BMI of 18.5-22.9 kg/m², which is normal. Five (9.6%) patients were found to have a BMI of 30.0-34.9 kg/m², which was severely obese. Two (3.8%) patients were morbidly obese, having a BMI of 35.0-39.9 kg/m². One (1.9%) patient had a BMI <18.5 kg/m² and thus was underweight.

The study published by Sarhan et al. and Sodhi et al. kept forward that a high BMI may contribute to the genesis of gallstone disease [23,24]. Due to the association between gallstones and obesity, the BMI was commonly used in older research to calculate the risk of developing gallstones. The ratio between fat distribution throughout the body and total body fat may impact gallstone development. Particularly obesity involving primarily the abdominal area is associated with several metabolic diseases, such as the development of gallstones, in both male and female patients. Higher BMI is supposed to be the most common major risk factor, which is preventable, contributing to the high prevalence of gallstones there. Alexander et al. study and Hayat et al. study discovered increased cholesterol levels, similar to what we found [25,26]. Several specialists have conjectured that certain types of mental stress can elevate blood cholesterol levels, which raises the likelihood of gallstone development [27]. Of all the patients, 52% with hypothyroidism and 50% with euthyroidism had a BMI of 25 to 30 and were obese [28]. Arun et al. [29] performed a study showing a result in contrast to the present study. The study showed more obese patients having cholelithiasis, which is inconsistent with the other studies (Table 8).

**Table 8:** Distribution of cholelithiasis patients in accordance with BMI in various studies

Study	Number of underweight patients / normal patients	Percentage of underweight patients/normal patients	Number of overweight patients/obese patients	Percentage of overweight patients/obese patients	Total
Present study	9	17.3	43	82.7	52
Arun et al. (2018) [29]	235	94	30	6	265
Sarhan et al. (2009) [23]	10	20	40	80	50
Raza et al. (2020) [28]	9	8.1	101	91.9	110

Association between thyroid status and BMI

Our study showed that in all the euthyroid patients, 21 (48.8%) patients were in the underweight/normal category whereas 22 (51.2%) patients were in the overweight category. Of all the patients with hypothyroidism, one (11.1%) patient was underweight/normal whereas eight (88.2%) patients were overweight. Raza et al. conducted a study and compared the distribution of cholelithiasis patients according to thyroid status and BMI [28]. The results of this study are consistent with the present study having more incidence of obesity in Euthyroid and Hypothyroid patients (Table 9).

**Table 9:** Association between thyroid status and BMI in various studies

Study	Thyroid status	Number of patients underweight/normal	Percentage of patients underweight/normal	Number of patients overweight/obese	Percentage of patients overweight/obese	Total
Present study	Euthyroidism	21	48.8	22	51.2	43
	Hypothyroidism	1	11.1	8	88.2	9
Raza et al. [28] (2020)	Euthyroidism	7	11.7	53	88.3	60
	Hypothyroidism	2	4	48	96	50

Distribution of cholelithiasis patients according to the thyroid status

Our study found that, out of the 52 patients, 43 (82.7%) were euthyroid, and nine (17.3%) had hypothyroidism. Of these nine hypothyroid patients, seven patients were subclinical whereas two patients showed overt disease. Völzke et al. found a connection between high TSH levels and gallstones that can be observed in sonography [30]. Kotwal et al. and Rinchen et al. found that 14.4% of the patients with hypothyroidism in Sikkim, India, also had cholelithiasis [31]. Yousif et al.'s study reached similar outcomes as those by Ibrahim et al. and Abdulbary et al. [32,33]. Arbab et al., in a randomized prospective trial, published that 16 out of 193 (8.16%) patients who had cholecystectomy done due to cholelithiasis also were diagnosed with hypothyroidism [34].

Ghadhban et al. found out through their study that 95 (92.2%) were found to be euthyroid, whereas eight of them (7.8%) were found to have subclinical hypothyroidism [18]. Hassan et al. performed a study that showed 12 cases of hypothyroidism out of the 138 patients included [33]. Ibrahim et al. and Raza et al. also had the same results of 52% and 45% of patients diagnosed with cholelithiasis having hypothyroidism, consistent with the findings of present study [28,32]. In our study, out of the nine patients having hypothyroidism, only two patients presented to us having symptoms of overt hypothyroidism (one patient had weight gain, moon face, loss of appetite, and constipation, whereas the other patient had constipation, generalized weakness, weight gain). Whereas a multi-center-based study denotes that the hypothyroidism had a prevalence of 10.95% in the general population [35]. The remaining seven patients had no clinically overt symptoms but had normal serum thyroid levels and raised TSH levels (subclinical hypothyroidism) (Table 10).

**Table 10:** Distribution of cholelithiasis patients in accordance to the thyroid status in various studies

Study	Number of patients having hypothyroidism	Percentage of patients having hypothyroidism	Number Euthyroid patients	Percentage Euthyroid patients	Total
Present study	9	17.3	43	82.7	52
Arbab et al. (2020) [34]	16	8.16	177	91.70	193
Ghadhban et al. (2019) [18]	8	7.8	95	92.2	103
Hassan et al. (2011) [33]	12	8	138	92	150
Ibrahim et al. (2014) [32]	52	52	48	48	100
Raza et al. (2020) [28]	50	45	60	55	110

## Conclusions

Our present study concludes that there is a correlation between cholelithiasis and hypothyroidism. The prevalence of cholelithiasis and hypothyroidism is higher in females. Most patients with cholelithiasis and hypothyroidism are elderly patients (>40 years). The study also denotes that the incidence of cholelithiasis and hypothyroidism is higher in obese patients (>25 kg/m^2 ^BMI). Our study showed that nine out of 52 patients with cholelithiasis had hypothyroidism. From our research, we have derived that there is a possibility of hypothyroidism in the patients diagnosed with cholelithiasis in 8.7%-30.8% of the cases.
